# A case of spontaneous basal ganglia hemorrhage with contralateral extension utilizing the canal of Gratiolet

**DOI:** 10.1186/s12883-023-03232-4

**Published:** 2023-05-12

**Authors:** Rahim Ismail, Derek D. George, Gurkirat S. Kohli, Muhammad W. Khan, Henry Z. Wang, Thomas K. Mattingly

**Affiliations:** 1 Department of Radiology, University of Rochester School of Medicine and Dentistry, Rochester, NY USA; 2 Department of Neurosurgery, University of Rochester School of Medicine and Dentistry, Rochester, NY USA; 3 Department of Neurology, University of Rochester School of Medicine and Dentistry, Rochester, NY USA

**Keywords:** Bilateral basal ganglia hemorrhage, Anterior commissure, Canal of Gratoilet, Hemorrhagic stroke, Case report

## Abstract

**Background:**

Intracranial hemorrhage accounts for 10–20% of stroke etiologies annually. Basal ganglia is the most common site for intracranial hemorrhage accounting for 50% of all cases. Bilateral spontaneous basal ganglia hemorrhages (BGH) are rare with few reported cases.

**Case presentation:**

We report an unusual case of a 69-year-old female who presented with a spontaneous bilateral basal ganglia hemorrhage secondary to a right BGH with contralateral extension through the anterior commissure (AC) utilizing the Canal of Gratiolet. Clinical course and imaging findings are discussed.

**Conclusions:**

To our knowledge, this is the first case to specifically detail the extension of spontaneous hemorrhage across the AC via the Canal of Gratiolet, and imaging findings provide a novel depiction of AC anatomy and fiber distribution in a clinical context. These findings may explain the mechanism behind this rare clinical entity.

## Background

Intracranial hemorrhage accounts for 10–20% of stroke etiologies annually [1]. Amongst intracranial hemorrhages, basal ganglia hemorrhage (BGH) are the most common site, estimated to comprise 50% of all cases [2]. The most common etiology is hypertensive hemorrhage followed by alcohol toxicity [3], although the exact etiology of these hemorrhages is often unknown. Spontaneous bilateral BGH occurs rarely, with only 60 instances having been reported in the literature [3]. We present a case demonstrating spontaneous bilateral BGH and discuss the anatomy in context of the pattern of hemorrhage on imaging.

## Case presentation/narrative

A 69-year-old female with a history of anxiety, hypothyroidism, and at least 32 pack-year smoking history with no pertinent family history who presented with sudden onset of headache and lethargy while away from home. The patient subsequently drove home with evidence of multiple collisions en route. She presented to the hospital where her initial blood pressure was 173/98 mmHg and physical exam was notable for mild encephalopathy, dysarthria, left facial droop, and left arm weakness. Patient was not on any anticoagulation or antiplatelet agents at the time of the event.

**Fig. 1:**
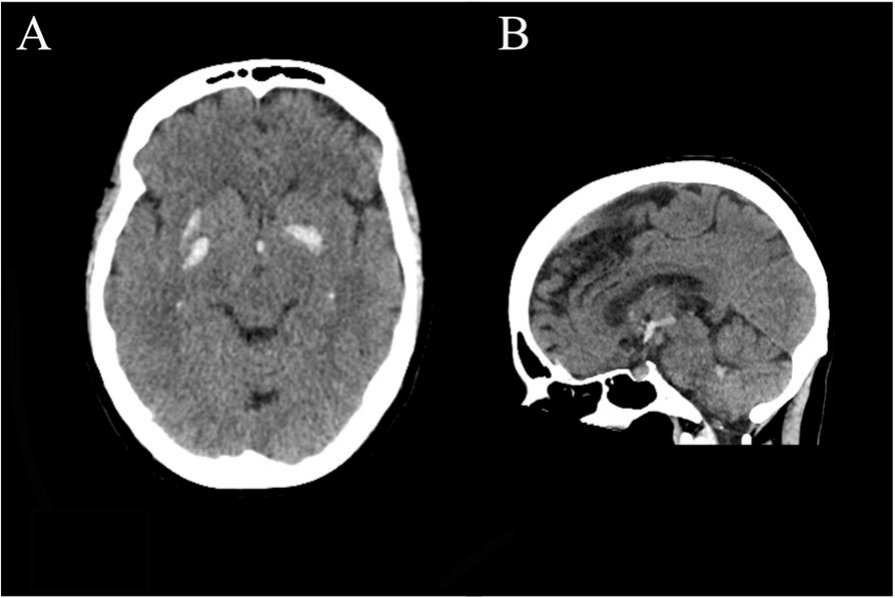
**A** Axial and (**B**) sagittal non-contrast computed tomography images show irregular hyperdensities along the right basal ganglia, contiguous along the anterior commissure (AC) and extending into the contralateral globus pallidus. Additional blood products are noted predominantly in the third ventricle and to a lesser extent in the fourth ventricle. Images acquired with a Philips Brilliance ICT 256 slice CT scanner

**Fig. 2:**
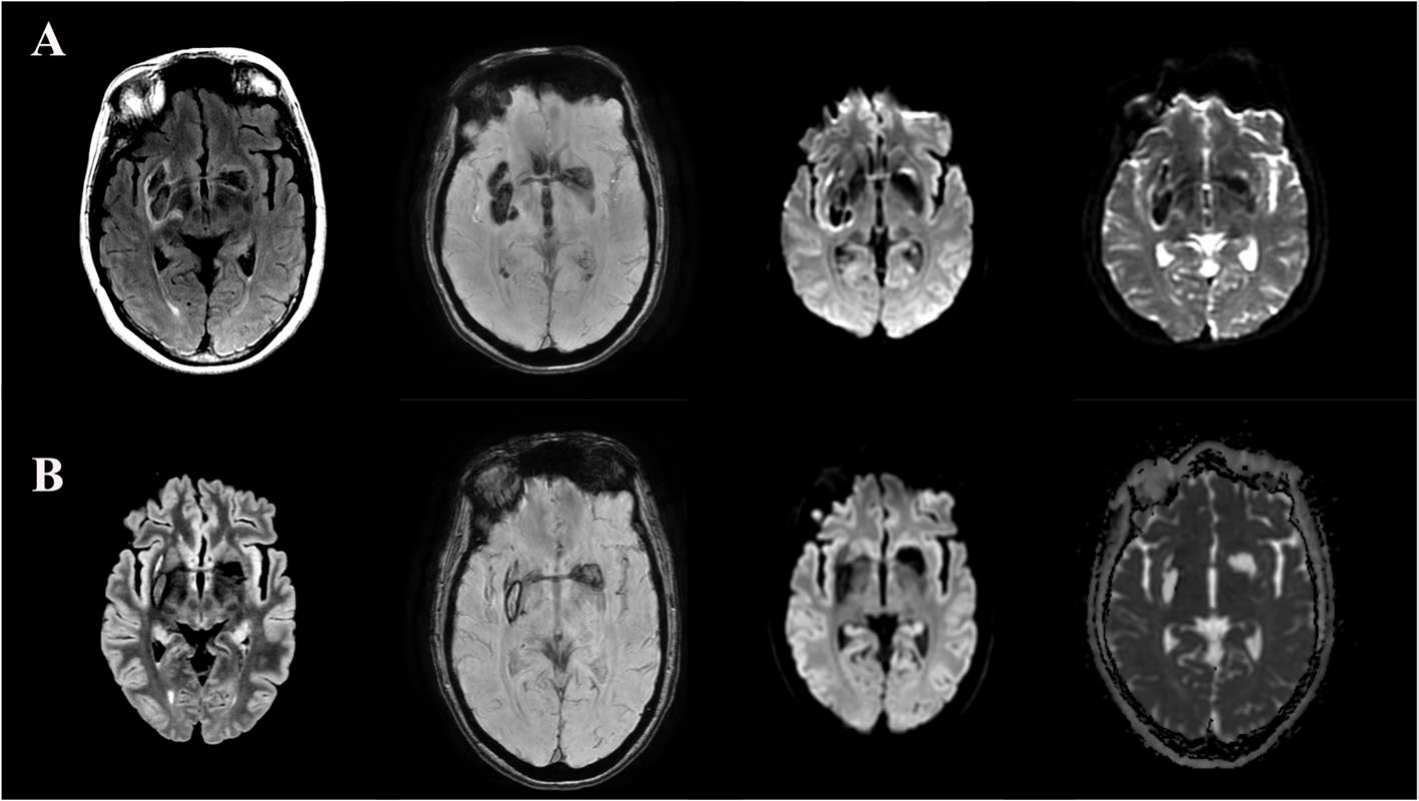
**A** Initial and (**B**) 7-month follow-up magnetic resonance images demonstrating the extent of right basal ganglia hemorrhage, contralateral extension and delineation of the AC tracts. At 7 months follow-up, the BGH and intraventricular hemorrhage had resolved, but residual susceptibility signal was appreciated along the extent of hemorrhage. From left to right, sequences presented are: T2 Fluid Attenuated Inversion Recovery (T2 FLAIR), Susceptibility-Weighted Angiography (SWAN), isotropic image/diffusion-weighted image (DWI), and apparent diffusion coefficient (ADC). Images acquired with a General Electric 750W 3 T MRI scanner

Non-contrast head computed tomography (CT) was obtained which demonstrated irregular hyperdensities along the right basal ganglia, contiguous along the anterior commissure (AC) and extending into the contralateral globus pallidus (Fig. 1). Intraventricular extension into the third ventricle, bilateral foramina of Monro, and fourth ventricle were noted. CT angiogram was also obtained without evidence of vascular malformation or aneurysm. Magnetic resonance imaging (MRI) was obtained which demonstrated hemosiderin staining and vasogenic edema along the white matter tract of the AC (Fig. 2). The distribution of edema seen on sagittal T2 images appeared restricted to a circular area of encased white matter. MR venogram was also obtained without evidence of cerebral venous occlusion. No vascular lesions were identified on subsequent catheter cerebral angiogram.

The patient was admitted and observed. Systolic blood pressure goals were less than 140 mmHg and the patient was resumed on home medications. Work-up was negative for coagulopathy or toxicologic etiology. At the time of discharge, the patient exhibited slightly decreased strength in the left upper extremity with a persistent pronator drift. At the time of last neurological exam 7 months post-hemorrhage, she exhibited a mild strength deficit in left elbow flexion and mild left upper extremity dysmetria. Follow-up imaging at 7 months demonstrated resolution of the BGH and intraventricular extension (Fig. 2).

## Discussion

We present a rare case of spontaneous bilateral BGH which likely occurred in the right basal ganglia and distributed to the contralateral basal ganglia across the anterior commissure via the Canal of Gratiolet.

The anterior commissure is a “handle-bar” shaped commissural white matter tract connecting bilateral frontal, temporal, and occipital lobes. It consists of both an anterior fiber bundle (or crus) connecting the olfactory and orbitofrontal cortices and a posterior fiber bundle. The posterior fiber bundle further divides into temporal and parietal occipital extensions**,** connecting the amygdala, middle temporal gyrus, and the occipital lobes [4, 5]. Its function remains largely unknown, although clinical data suggest a possible role in visual memory [6]. Clinically, the AC has been primarily studied as an anatomical landmark for stereotactic neurosurgery. However, when considered as a commissural fiber tract more generally, there are numerous pathologies that may be involved. Dissemination of gliomas and shear injury from trauma can affect commissural fibers. Although this is classically seen along the corpus callosum, selective invasion of the AC has been reported [4]. Additionally, traumatic injury has been described along the AC in human and animal studies [7–9] likely secondary to the known propensity of white matter tracts to shear in acceleration/deceleration injury.

At the time of this report, the authors are not aware of any spontaneous BGH with contralateral involvement secondary to communication via the AC. Only a few traumatic cases with similar radiographic findings have been published. Beers et al. reported on presumed fat embolism-associated intracranial hemorrhage after the patient was struck by a car [10]. Axial CT of the head demonstrated a contiguous hyperdensity in the region of the AC with hemorrhage in bilateral globi pallidi. The authors report “the fat embolism may have resulted from vascular stasis brought on by shearing injury to the anterior commissure.” Thus, the origin of the hemorrhage was presumed to be secondary to accumulation of local microhemorrhages. Mistry also documented a case of traumatic hemorrhage extending across the AC in a 19-year-old male [11].

Our case demonstrates bilateral hemorrhage likely originating in the right hemisphere. Initial CT imaging demonstrated lobulated hyperdensities in bilateral basal ganglia and MRI demonstrated hemosiderin staining and vasogenic edema in the right putamen and globus pallidus which tracked along the posterior limb of the AC via the Canal of Gratiolet into the contralateral basal ganglia. Additional intraventricular extension was noted, with the greatest extent of hemorrhage in the third ventricle. Follow-up imaging at 7 months demonstrated resolution of the BGH and intraventricular extension. Susceptibility-weighted Angiography (SWAN) revealed persistent susceptibility signal in the AC and Canal of Gratiolet. While our team did consider investigating alterations in AC morphology with diffusion tensor tractography, we concluded this would not be useful due to hemosiderin artifact. Another limitation of our study is that we did not formally investigate visual memory function in our patient. Thus, we may have missed subtle clinical findings of AC fiber disruption according to its presumed function in visual memory processing [6].

We acknowledge the difficulty in establishing a definitive mechanism for bilateral basal ganglia hemorrhage in our case. Without serial imaging throughout the patient’s hospital stay, spontaneous bilateral BGH cannot be definitively refuted. However, both clinical and radiological features suggest right-sided nidus of hemorrhage with subsequent contralateral extension. Clinically, the patient's symptoms were left sided (e.g. left facial droop and left arm weakness), suggesting initial and more severe neuronal disruption in the right basal ganglia. Radiographically, the pattern of hemosiderin staining noted on MRI is observed to be more cavitary on the right, suggestive of a focal/disruptive source of hemorrhage (e.g. vascular rupture). In contrast, the left-sided hemosiderin staining appears diffuse, suggestive of spread along the fascicles of the AC. Additionally, the degree of edema surrounding the hemorrhages is asymmetric: A greater degree of cytotoxic edema on the right suggests greater duration or degree of insult, further suggesting right-sided hemorrhage nidus. Regarding the most likely etiology of our patient’s hemorrhage, elevated blood pressure on presentation along with CT- and catheter angiography showing no vascular malformation or aneurysm therefore makes hypertensive microangiopathy most likely. This etiology is concordant with the published literature showing hypertension to be the most likely etiology of bilateral BGH [3, 12].

**Fig. 3:**
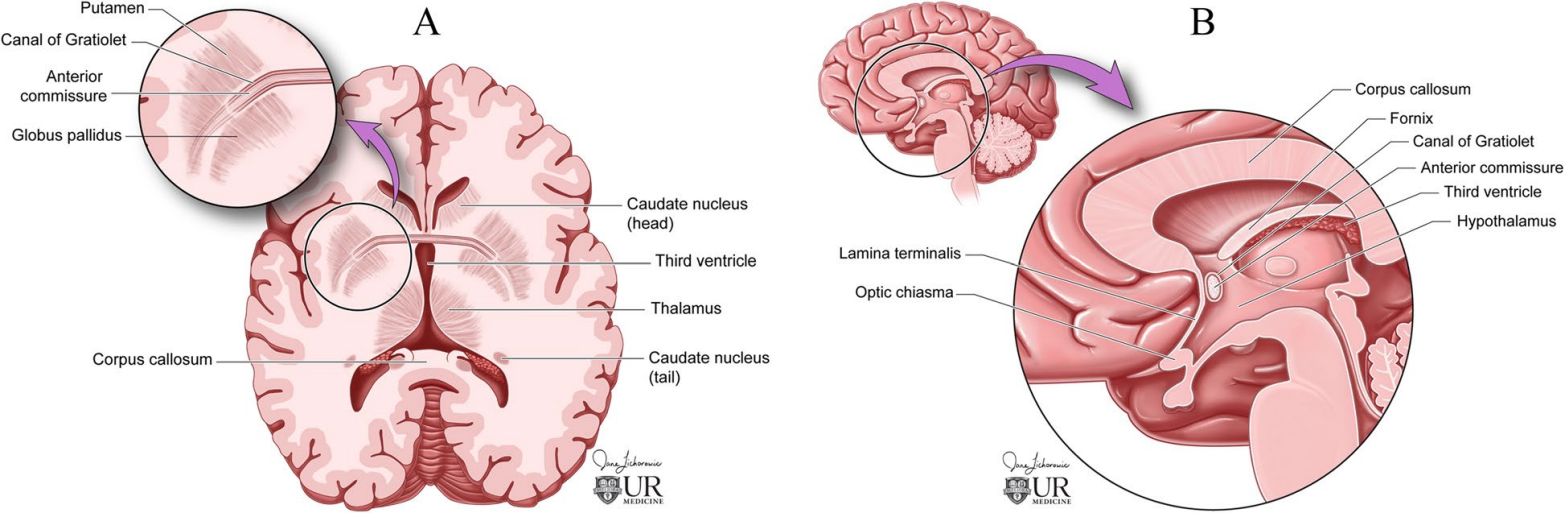
**A** Axial and (**B**) sagittal illustrations of the Canal of Gratiolet (CoG) among surroundings structures. Note the start of the CoG surrounding the posterior fascicle of the AC situated between the putamen and the globus pallidus before extending anteromedially to envelope the AC toward the midline

In anatomical dissections, the canal of Gratiolet has been described as a thin wrapping of grey matter covering the lateral trajectory of the posterior white matter tracts of the AC as it runs parallel and deep to the uncinate fasciculus [13–16]. Medially, the white matter of the anterior commissure is encased by the striatum, with the lateral extension (Canal of Gratiolet) formed between the structures of the lentiform nucleus (Fig. 3). This structural geography and pattern of myelin segregation were reflected in our case by the pattern of hemosiderin staining and focal white matter edema. Additionally, our case demonstrated even further lateral extension than previously demonstrated by Beers et al., with continuation of hemorrhage along the posterior fascicle (Fig. 2), further delineating the fiber anatomy of the AC. Interestingly, our case exhibited intraventricular extension of the hemorrhage into the third ventricle, bilateral foramina of Monro, and the fourth ventricle, with the thickest density of blood products in the third ventricle. This hemorrhage pattern suggests extension of blood products intraventricularly via the exposed surface of the anterior commissure at the anterior surface of the third ventricle [17]. Due to the rarity of this clinical entity, the incidence of bilateral BGH with intraventricular extension is unknown. In one case of spontaneous bilateral BGH, intraventricular extension into the right frontal horn was observed on non-contrast CT and MRI [18]. In another case of bilateral thalamic hemorrhage, layering blood products were observed in the dependent portion of the left lateral ventricle on non-contrast CT [19]. These cases demonstrate that intraventricular extension can occur with these hemorrhages. However, we believe our case to be the first to demonstrate primary extension of bilateral BGH into the third ventricle via the exposed surface of the AC.

This case represents a rare example of hemorrhage extension across the AC and is the first description to our knowledge of extension of spontaneous BGH across the AC via the Canal of Gratiolet. Because of the rarity of spontaneous bilateral basal ganglia hemorrhage, the exact mechanism by which bilateral hemorrhages occur is unknown. Our case provides a possible anatomic explanation: a primary unilateral hemorrhage expands across a commissural white matter tract into contralateral counterpart structures. Such a phenomenon has previously been reported within the corpus callosum, although this was in the context of a hemorrhagic metastatic lesion [20]. In our case, the right globus pallidus was the presumed primary hemorrhage site, with subsequent spread within the Canal of Gratiolet contralaterally. Our case nicely demonstrates the macroscopic anatomy of the anterior commissural fibers as correlated with hemosiderin staining and T2 hyperintensities visualized on MRI, as well as the AC as a structure delimiting the anterior wall of the third ventricle. Imaging findings thus provide a unique depiction of AC anatomy and fiber distribution in a clinical context and support the mechanistic hypothesis of bilateral BGH originating via commissural extension. Our study is limited to a signal patient, and is therefore useful for hypothesis generation regarding a possible mechanism for appearance of bilateral BGH. Further studies and reports may further elucidate this possible mechanism of hemorrhage spread.

### Patient perspective

Our patient recounts their initial experiences with hemorrhage development: The patient was at the veterinarian’s office in the process of euthanizing her dog and states: "I felt a ripping pain at the back of my head that seemed to envelope both sides. I suddenly felt very nauseous." The patient headed back to the car, lay back in the car seat until they had pain relief and felt stable enough to drive home. "The severe pain lasted about 20 min". When asked what our patient did for the pain, they said they “took an Advil [ibuprofen].” As the pain became a bit more manageable, the patient started the 30-min drive back to their residence. "I do not remember much of the drive." The patient was visited by family later that evening who urged the patient to go to the hospital for evaluation. During our initial conversation with the patient’s family member, the family member endorsed seeing scratches and bumps on the car; “… definitely hit some things on the drive back…".

Regarding the care our patient received, they remarked: “They did all kinds of tests on me and took a bunch of pictures of my head and blood vessels with MRIs and an angiogram. It felt like a lot. Everyone was confused about why my bleed happened. They still don’t know exactly what caused it.”

## Data Availability

Data sharing is not applicable to this article as no datasets were generated or analyzed during the current study. All data generated and analyzed during this study are included in this article.

## Abbreviations

BGH: Basal ganglia hemorrhage
CT: Computed tomography
AC: Anterior commissure
MRI: Magnetic resonance imaging
SWAN: Susceptibility-weighted angiography
T: Tesla
T2 FLAIR: T2 Fluid Attenuated Inversion Recovery
DWI: Diffusion-weighted image/isotropic image
ADC: Apparent diffusion coefficient
CoG: Canal of Gratiolet
